# Ferulic Acid, Pterostilbene, and Tyrosol Protect the Heart from ER-Stress-Induced Injury by Activating SIRT1-Dependent Deacetylation of eIF2α

**DOI:** 10.3390/ijms23126628

**Published:** 2022-06-14

**Authors:** Kévin Monceaux, Mélanie Gressette, Ahmed Karoui, Julie Pires Da Silva, Jérôme Piquereau, Renée Ventura-Clapier, Anne Garnier, Mathias Mericskay, Christophe Lemaire

**Affiliations:** 1 Faculté de Pharmacie, UMR-S 1180, INSERM, Université Paris-Saclay, 5 Rue J-B Clément, 92296 Châtenay-Malabry, France; kevin.monceaux.km@gmail.com (K.M.); melanie.gressette@universite-paris-saclay.fr (M.G.); ahmed.karoui@universite-paris-saclay.fr (A.K.); julie.piresdasilva@gmail.com (J.P.D.S.); jerome.piquereau@universite-paris-saclay.fr (J.P.); renee.ventura@universite-paris-saclay.fr (R.V.-C.); anne.garnier@universite-paris-saclay.fr (A.G.); mathias.mericskay@inserm.fr (M.M.); 2 Inserm, UMR-S 1180, Université Versailles St-Quentin, Université Paris-Saclay, 92296 Châtenay-Malabry, France

**Keywords:** UPR, sirtuin 1, phenolic compounds, cardioprotection, apoptosis

## Abstract

Disturbances in Endoplasmic Reticulum (ER) homeostasis induce ER stress, which has been involved in the development and progression of various heart diseases, including arrhythmias, cardiac hypertrophy, ischemic heart diseases, dilated cardiomyopathy, and heart failure. A mild-to-moderate ER stress is considered beneficial and adaptative for heart functioning by engaging the pro-survival unfolded protein response (UPR) to restore normal ER function. By contrast, a severe or prolonged ER stress is detrimental by promoting cardiomyocyte apoptosis through hyperactivation of the UPR pathways. Previously, we have demonstrated that the NAD^+^-dependent deacetylase SIRT1 is cardioprotective in response to severe ER stress by regulating the PERK pathway of the UPR, suggesting that activation of SIRT1 could protect against ER-stress-induced cardiac damage. The purpose of this study was to identify natural molecules able to alleviate ER stress and inhibit cardiomyocyte cell death through SIRT1 activation. Several phenolic compounds, abundant in vegetables, fruits, cereals, wine, and tea, were reported to stimulate the deacetylase activity of SIRT1. Here, we evaluated the cardioprotective effect of ten of these phenolic compounds against severe ER stress using cardiomyoblast cells and mice. Among the molecules tested, we showed that ferulic acid, pterostilbene, and tyrosol significantly protect cardiomyocytes and mice heart from cardiac alterations induced by severe ER stress. By studying the mechanisms involved, we showed that the activation of the PERK/eIF2α/ATF4/CHOP pathway of the UPR was reduced by ferulic acid, pterostilbene, and tyrosol under ER stress conditions, leading to a reduction in cardiomyocyte apoptosis. The protection afforded by these phenolic compounds was not directly related to their antioxidant activity but rather to their ability to increase SIRT1-mediated deacetylation of eIF2α. Taken together, our results suggest that ferulic acid, pterostilbene, and tyrosol are promising molecules to activate SIRT1 to protect the heart from the adverse effects of ER stress.

## 1. Introduction

The rough endoplasmic reticulum (ER) is a central organelle involved in the synthesis, folding, and quality control of transmembrane and secretory proteins. The impairment of ER homeostasis, caused by physiological or pathological conditions such as calcium homeostasis perturbations or oxidative stress, results in the accumulation of unfolded and misfolded proteins inside the ER lumen, a condition known as ER stress [1]. With the aim to restore ER proteostasis and homeostasis, an adaptive mechanism called the unfolded protein response (UPR) is triggered in response to ER stress. The UPR is initiated by the dissociation of the master ER resident chaperone GRP78/BiP from the three ER stress sensors PERK, ATF6, and IRE1, thereby allowing their activation. When activated, these sensors initiate a transcriptional and translational response that gives rise to a decrease in protein neosynthesis through the phosphorylation of the translation initiation factor eIF2α, the transcription of UPR target genes (e.g., *XBP1*, *ATF4*), and the increase in the expression of genes encoding ER chaperones, proteins involved in the ER-associated protein degradation pathway (ERAD), and proteins of the autophagy pathway. However, when the ER is under chronic or excessive stress and the UPR fails to restore ER homeostasis, apoptosis is triggered to eliminate damaged cells. This can be correlated with the pathogenesis of a wide range of human diseases [2].

Numerous animal models and increasing human studies have shown that ER stress is involved in the development and progression of a large number of heart diseases including ischemic heart diseases, arrhythmias, cardiac hypertrophy, and heart failure [3]. Currently, a mild-to-moderate ER stress is considered beneficial for heart function by restoring ER homeostasis and promoting survival. For example, the overexpression of several UPR regulators, including GRP78/BiP and ATF6, protects cardiomyocytes from cell death induced by ischemia/reperfusion [4,5]. Conversely, a severe or prolonged ER stress is known to be deleterious by promoting cardiomyocyte death [3,6]. Mice knocked out for CHOP, the main activator of the apoptotic arm of the UPR, develop less cardiac dysfunction compared to wild-type mice in response to transverse aortic constriction (TAC) [7]. Thus, modulating the UPR by promoting adaptative and pro-survival pathways and/or inhibiting damaging pro-apoptotic pathways is a potential therapeutic prospect for the treatment of cardiovascular diseases associated with ER stress.

As a stress sensor, the NAD^+^-dependent deacetylase Sirtuin 1 (SIRT1) has been shown to be cardioprotective in response to a number of cardiac insults including ischemia, pressure overload, cardiac hypertrophy, or aging [8]. The protective effects of SIRT1 rely on its ability to deacetylate various substrates and transcription factors such as p53, FOXOs, and PGC1-α [9]. Recently, we have shown that SIRT1 protects the heart from severe ER stress by deacetylating eIF2α, regulating the PERK pathway of the UPR [10], and promoting autophagy through activation of the eEF2K/eEF2 pathway [11]. Therefore, the activation of SIRT1 appears as a potentially valuable therapeutic strategy to treat cardiac pathologies associated with severe ER stress. Substantial research efforts devoted to the identification of SIRT1 activators have led to the discovery of both natural and synthetic sirtuin-activating compounds (STACs), a variety of small molecules shown to facilitate SIRT1 activation. Additionally, bioactive peptides, derived from ovotransferrin, were recently shown to stimulate SIRT1 activity through the NAMPT/NAD^+^ axis and to protect cells from drug-induced cytotoxicity [12,13]. Among the natural molecules, resveratrol, a phytochemical polyphenol found in many plants including grapes and grape products (wine, juice), peanuts, and berries, was the first identified activator of SIRT1 [14]. Subsequently, other natural molecules of the phenolic compounds’ superfamily, found in plants and in our diet, were suggested to activate SIRT1 [15,16,17,18,19,20]. Several of these phytochemicals have shown promising beneficial effects on cardiac function owing mostly to their antioxidant, anti-inflammatory, and anti-apoptotic effects [21,22,23].

**Figure 1:**
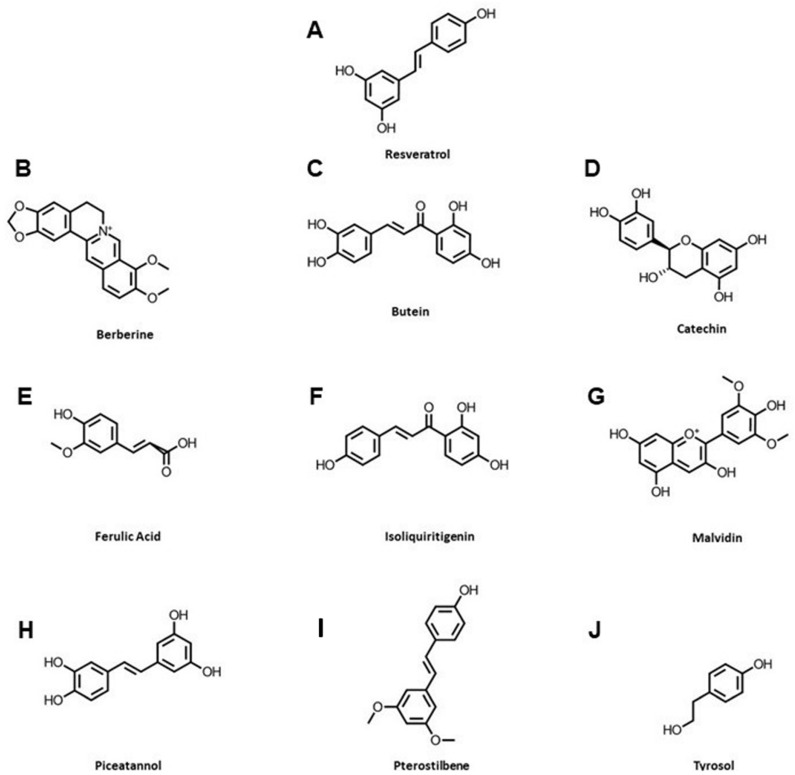
Chemical structures of phenolic compounds used in this study. Resveratrol is mainly found in grapes and berries (**A**), Berberine in barberry fruits and turmeric (**B**), Butein in broad beans (**C**), Catechin in green tea, cocoa, and apricots (**D**), Ferulic acid in cereals such as rice, wheat, and oats (**E**), Isoliquiritigenin in liquorice (**F**), Malvidin (**G**), Piceatannol (**H**), and Pterostilbene (**I**) in grapes and blueberries, and Tyrosol in olives and grapes (**J**).

The current study was aimed at identifying bioactive natural molecules with the ability to alleviate ER stress and inhibit cardiomyocyte cell death. The ten phenolic compounds resveratrol, berberine, butein, catechin, ferulic acid, isoliquiritigenin, malvidin, piceatannol, pterostilbene, and tyrosol were demonstrated to protect the heart from various stresses and are independently suggested to activate SIRT1, although this is seen primarily in non-cardiovascular cell models (Figure 1) [17,20,24,25,26,27,28]. We thus tested the ability of these natural phytonutrients to protect the heart from severe ER stress in in vitro (H9c2 cells and isolated adult rat ventricular myocytes (ARVM)) and in vivo (C57BL/6J mice) models. After screening the molecules’ ability to elicit cardioprotection against severe ER stress, further analysis was carried out to determine the mechanisms involved and whether this cardioprotection may be attributed to an activation of SIRT1.

## 2. Results

### 2.1. Determination of the Effect of the Phytochemicals on Cardiac Cell Viability

**Figure 2:**
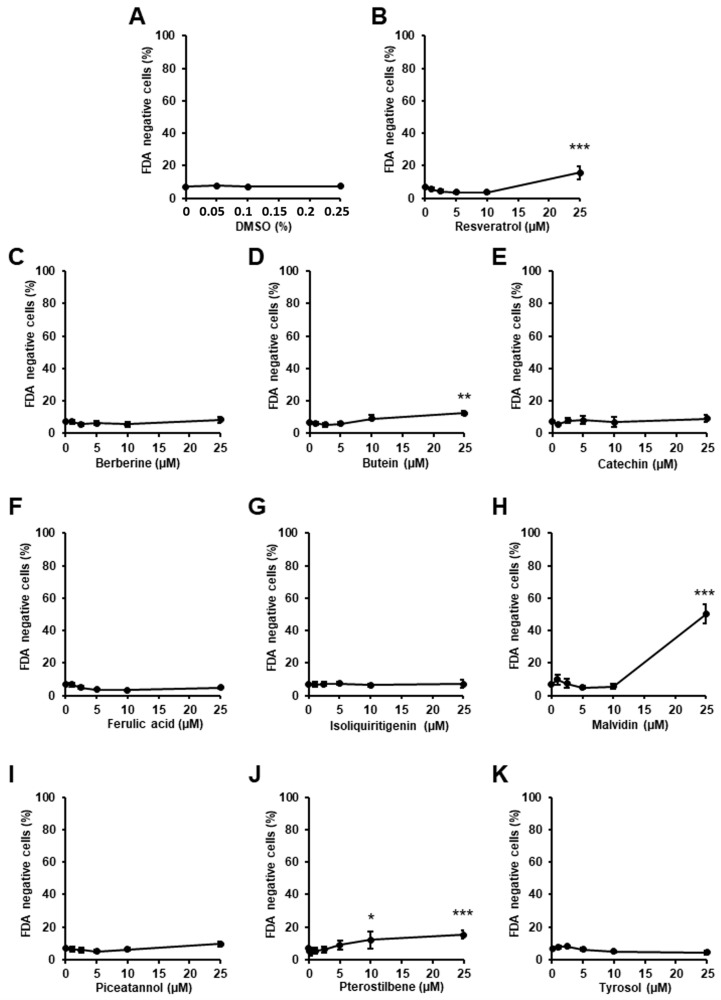
Effect of phenolic compounds on cardiac cell viability. Cell viability was assessed in H9c2 cells by flow cytometry using FDA assay following 48 h treatment with DMSO (**A**, *n* = 3), resveratrol (**B**, *n* = 5), berberine (**C**, *n* = 5), butein (**D**, *n* = 5), catechin (**E**, *n* = 3), ferulic acid (**F**, *n* = 4), isoliquiritigenin (**G**, *n* = 6), malvidin (**H**, *n* = 4), piceatannol (**I**, *n* = 5), pterostilbene (**J**, *n* = 4), or tyrosol (**K**, *n* = 5). FDA is cleaved into green fluorescein by intracellular esterases present in living cells. All non-FDA-fluorescing cells are considered dead. The percentages of dead cells are presented (Cell death (%)). Values represent mean ± S.E.M. * *p* < 0.05, ** *p* < 0.01, *** *p* < 0.001 vs. control (0 µM).

To eliminate the potential toxic effects of the ten selected phenolic compounds (Figure 1), we first evaluated the effect of each molecule on the viability of cardiac H9c2 cells by flow cytometry using fluorescein diacetate (FDA) viability assay (Figure 2). Cells were treated for 48 h with increasing concentrations of each molecule ranging from 0 to 25 µM, and the percentage of FDA negative cells, corresponding to the percentage of dead cells, was analyzed. DMSO, which was used as a vehicle, did not show any toxicity to H9c2 cells at the concentrations used in this study (Figure 2A). Resveratrol, the most-studied SIRT1 activator, its dimethylated analog pterostilbene, and butein and malvidin increased cardiac cells’ death above 10 µM (Figure 2B,D,H,J). The other compounds, i.d. berberine, catechin, ferulic acid, isoliquiritigenin, piceatannol, and tyrosol, did not induce cell death at the tested concentrations (Figure 2C,E–G,I,K). According to these results, the phenolic compounds were used at concentrations below 10 µM for all further experiments on cardiac cells.

### 2.2. Ferulic Acid, Pterostilbene, and Tyrosol Protect Adult Cardiomyocytes from Cell Death Induced by Severe ER Stress

**Figure 3:**
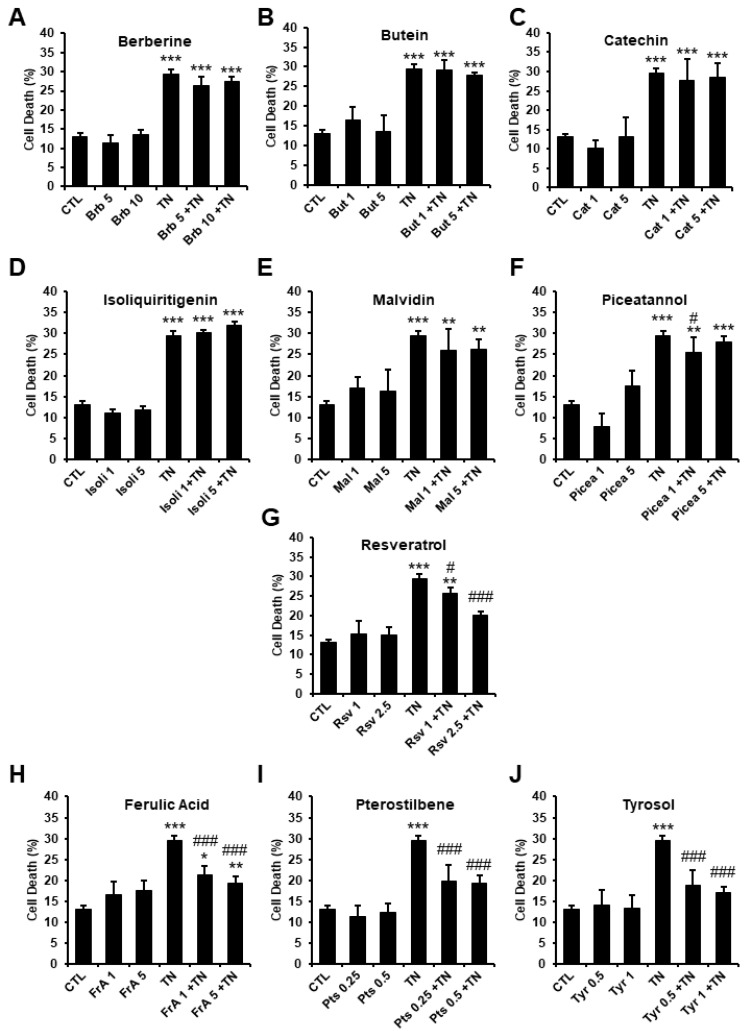
Effect of phenolic compounds on ER-stress-induced cardiomyocyte cell death. Cell death was evaluated by fluorescence microscopy using FDA assay. The FDA is cleaved into green fluorescein by intracellular esterases present in living cells. All non-FDA-fluorescing cells are considered dead. The percentages of dead cells are presented (Cell death (%)). Adult rat ventricular myocytes (ARVM) were treated for 24 h with tunicamycin (TN, 10 µg/mL) after 1 h pre-treatment with berberine (**A**, *n* = 3), butein (**B**, *n* = 3), catechin (**C**, *n* = 5), isoliquiritigenin (**D**, *n* = 3), malvidin (**E**, *n* = 4), piceatannol (**F**, *n* = 4), resveratrol (**G**, *n* = 3), ferulic acid (**H**, *n* = 6), pterostilbene (**I**, *n* = 5), or tyrosol (**J**, *n* = 5). Concentrations used are expressed in µM. Values represent mean ± S.E.M. * *p* < 0.05, ** *p* < 0.01, *** *p* < 0.001 vs. control; # *p* < 0.05, ### *p* < 0.001 vs. TN.

To evaluate the effect of the phenolic compounds on ER-stress-induced cell death in cardiomyocytes, adult rat ventricular myocytes (ARVM) were pre-treated or not with the different molecules for 1 h, then severe ER stress was induced with the well-known ER stressor tunicamycin (TN, 10 µg/mL). The percentage of dead cells was measured by fluorescence microscopy using FDA viability assay. As expected, after 24 h of treatment with TN, the percentage of dead cells significantly increased compared to the control (Figure 3). The phytochemicals alone did not induce cardiomyocyte death at the concentrations tested, which is consistent with the results obtained in H9c2 cells (Figure 2). Among the molecules studied, berberine, butein, catechin, isoliquiritigenin, malvidin, and piceatannol did not significantly modify the mortality induced by TN treatment (Figure 3A–F). By contrast, resveratrol (Figure 3G), as well as ferulic acid, pterostilbene, and tyrosol (Figure 3H–J) significantly protected ARVM from ER stress-induced cell death, as revealed by the significant decrease in the percentage of FDA negative cells (dead cells) observed when ARVM were incubated with TN plus these molecules, compared to TN alone. Interestingly, the protective effect of pterostilbene and tyrosol occurred at concentrations that were respectively 5 (0.5 µM) and 2.5 (1 µM) fold lower than that required to observe protection with resveratrol (2.5 µM). Taken together, our results reveal that ferulic acid, pterostilbene, and tyrosol protect adult cardiomyocytes from ER-stress-induced cell death.

### 2.3. Ferulic Acid, Pterostilbene, and Tyrosol Protect the Heart from Damage Induced by Severe ER Stress

**Figure 4:**
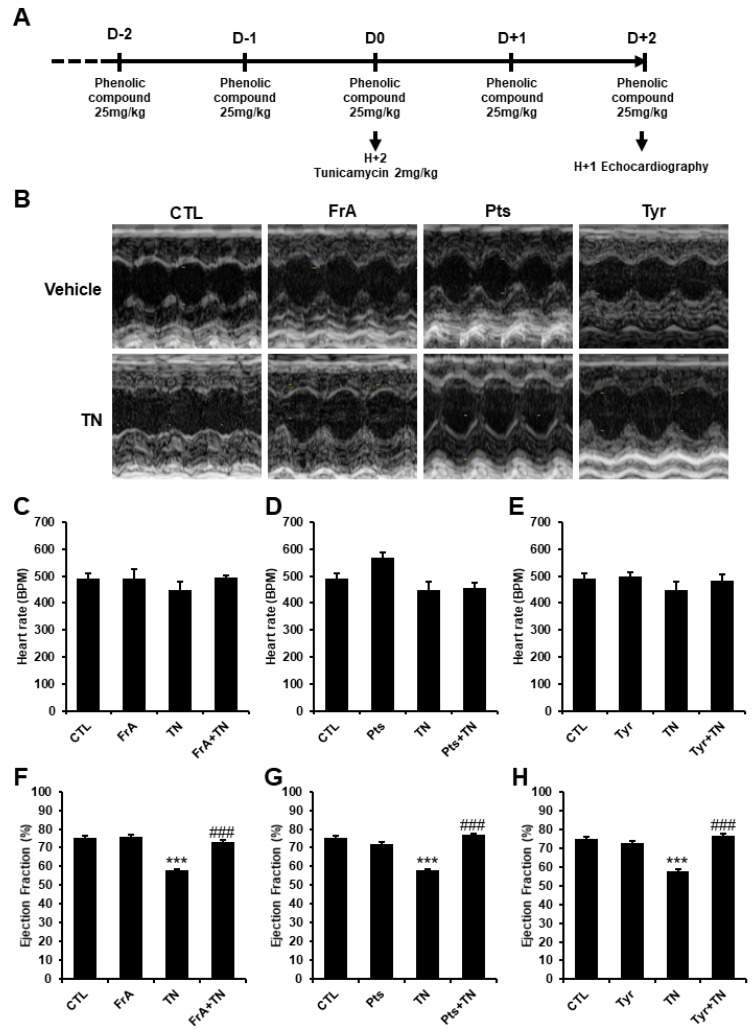
Ferulic acid, pterostilbene, and tyrosol protect the heart from ER-stress-induced dysfunction. Male C57BL/6J mice (12 weeks old) were injected i.p. every day for 5 days with the vehicle or 25 mg/kg of ferulic acid (FrA, **C**,**F**), pterostilbene (Pts, **D**,**G**), or tyrosol (Tyr, **E**,**H**). At day 3, 1 h after phytochemical injection, mice were given 2 mg/kg tunicamycin (TN) by intraperitoneal injection to induce ER stress. Cardiac function was analyzed 48 h after TN injection by echocardiography (**A**). Representative images obtained by transthoracic echocardiography (**B**). Heart rate (**C**–**E**) and ejection fraction (**F**–**H**) are presented. Values represent mean ± S.E.M. (*n* = 5) *** *p* < 0.001 vs. CTL; ### *p* < 0.001 vs. TN.

To investigate the effects of ferulic acid, pterostilbene, and tyrosol on cardiac function in the context of severe ER stress in vivo, adult male C57BL6/J mice were injected for 5 days with one of the phenolic compounds (25 mg/kg body weight, i.p.). ER stress was induced on the 3rd day by TN injection (2 mg/kg body weight, i.p.), and the cardiac function was evaluated by echocardiography on day 5, 2 h after the last phytochemical injection (Figure 4A,B). Mice treated with ferulic acid, pterostilbene, or tyrosol alone did not exhibit modifications in their heart rate or ejection fraction, indicating that these natural compounds are unlikely to be toxic in vivo at this concentration (Figure 4). In agreement with our previous studies [10,11], when severe ER stress was induced, the ejection fraction was reduced to 57.8 ± 2.8% compared to 75.2 ± 1.6% in control mice (Figure 4F–H), without changing their heart rate (Figure 4C–E). Mice treated with ferulic acid, pterostilbene, and tyrosol were totally protected from ER-stress-induced cardiac alterations and exhibited ejection fractions and fractional shortenings which were comparable to that of untreated mice (Figure 4F–H and Supplementary Table S2). These results highlight the powerful cardioprotective effect of these molecules against severe ER stress in vivo.

### 2.4. Ferulic Acid, Pterostilbene, and Tyrosol Protect Cardiac Cells from Apoptosis Induced by Severe ER Stress

**Figure 5:**
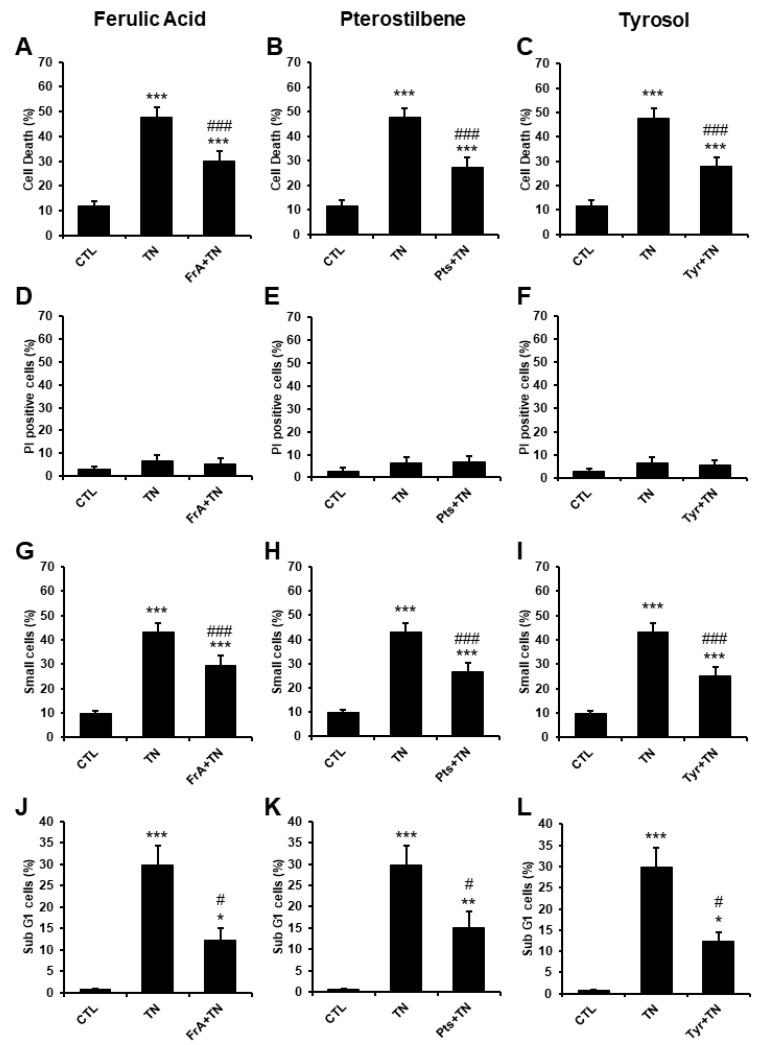
Ferulic acid, pterostilbene, and tyrosol protect cardiac cells from apoptosis induced by severe ER stress. H9c2 cells were pre-treated for 1 h with ferulic acid (FrA, 5 µM), pterostilbene (Pts, 0.5 µM), or tyrosol (Tyr, 1 µM), and then incubated for 48 h with tunicamycin (10 µg/mL). Cell death was analyzed by flow cytometry using FDA assay (**A**–**C**, *n* = 12). FDA is cleaved into green fluorescein by intracellular esterases present in living cells. All non-FDA-fluorescing cells are considered dead. The percentages of dead cells are presented (Cell death (%)). Necrosis was evaluated by measuring plasma membrane permeability by flow cytometry using propidium iodide (**D**–**F**, *n* = 3). Apoptosis was analyzed by flow cytometry by measuring cell shrinkage (small cells) (**G**–**I**, *n* = 12) and DNA fragmentation (Sub G1 cell population) (**J**–**L**, *n* = 4). Values represent mean ± S.E.M. * *p* < 0.05, ** *p* < 0.01, *** *p* < 0.001 vs. control; # *p* < 0.05, ### *p* < 0.001 vs. TN.

To further investigate the molecular mechanisms involved in the cardioprotective effect of ferulic acid, pterostilbene, and tyrosol, the cardiac H9c2 cell line was used. H9c2 cells were pre-treated for 1 h with or without ferulic acid (5 µM), pterostilbene (0.5 µM), or tyrosol (1 µM) before induction of severe ER stress by TN. After 48 h, cell death was analyzed by flow cytometry using FDA assay. As expected, TN increased H9c2 cell death (47.8 ± 3.8%) in comparison with the controls (11.8 ± 2%) (Figure 5A–C). Ferulic acid, pterostilbene, and tyrosol significantly protected H9c2 cells from cell death induced by TN treatment with approximately 40–50% of protection, corroborating the results obtained in ARVM (Figure 3H–J). We previously showed that tunicamycin triggers a severe ER stress associated with apoptosis induction [10]. We thus wondered whether ferulic acid, pterostilbene, and tyrosol protected cardiac cells from ER stress-induced cell death through inhibition of the apoptotic process. The integrity of the plasma membrane, which is maintained during apoptosis but not during necrosis, was measured by flow cytometry using the membrane-impermeant probe propidium iodide (PI). No increase in the percentage of PI positive cells was observed either with TN alone or with the phytochemicals plus TN (Figure 5D–F), indicating that necrosis was not induced in our cardiac model of severe ER stress. Besides, TN significantly increased the percentage of small cells (Figure 5G–I) and cells in the SubG1 population (Figure 5J–L), which are indicators of cell shrinkage and DNA fragmentation, respectively, two characteristic features of apoptosis. Ferulic acid, pterostilbene, and tyrosol reduced the percentage of small cells and cells with DNA fragmentation induced by ER stress, indicating that these natural phenolic compounds protect cardiac cells from apoptosis induced by severe ER stress.

### 2.5. The Cardioprotective Effect of Ferulic Acid, Pterostilbene, and Tyrosol Do Not Seem to Be Directly Related to Their Antioxidant Activity

**Figure 6:**
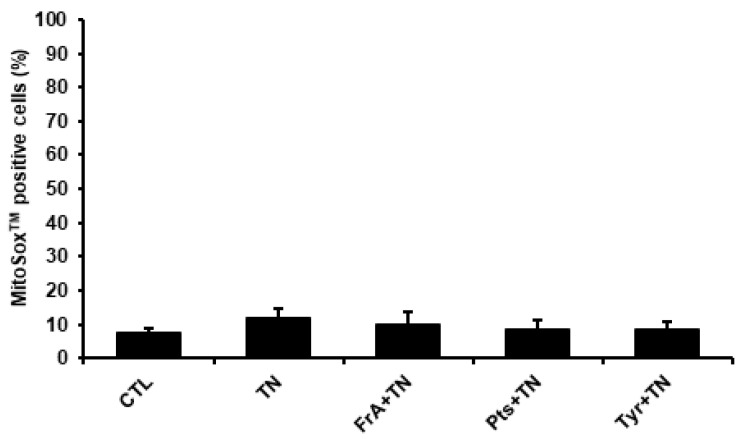
The antioxidant activity of ferulic acid, pterostilbene, and tyrosol do not seem to directly account for their cardioprotective effects. H9c2 cells were pre-treated for 1 h with ferulic acid (FrA, 5 µM), pterostilbene (Pts, 0.5 µM), or tyrosol (Tyr, 1 µM) and then treated for 24 h with tunicamycin (TN, 10 µg/mL). Accumulation of the mitochondrial superoxide anion O_2_^−^ was measured by flow cytometry using the MitoSOX^TM^ Red probe. Values represent mean ± S.E.M. (*n* = 5).

The cardioprotective effects of phenolic compounds have been largely attributed to their well-recognized antioxidant properties [22,23]. Therefore, to determine whether ferulic acid, pterostilbene, and tyrosol exert their cardioprotective effect against severe ER stress through their antioxidant activity, the production of mitochondrial superoxide anion and cellular H_2_O_2_ were analyzed in H9c2 cells by flow cytometry using MitoSOX™ Red and H2DFCDA probes, respectively. After 24 h of treatment, TN did not significantly modify the percentage of cells with an elevated content of mitochondrial superoxide anions (Figure 6, MitoSOX™ positive cells) or cellular H_2_O_2_ (Supplemental Figure S1A, DCF positive cells), suggesting that TN-induced apoptosis is not linked to an early increase in ROS production in cardiac cells. Accordingly, the addition of ferulic acid, pterostilbene, or tyrosol did not further change the level of mitochondrial superoxide anions in response to TN. As a positive control of ROS production, H9c2 cells were also treated with H_2_O_2_ (250 µM)_,_ a well-known generator of reactive oxygen species. As shown in supplemental Figure S1, H_2_O_2_ increased both ROS production (Figure S1B) and cell death in H9c2 cells (Figure S1C). In addition, by decreasing the level of mitochondrial superoxide anions, the antioxidant N-acetylcysteine decreased the percentage of H_2_O_2_-induced cell death_._ Thus, using flow cytometry, we were able to measure ROS production and a scavenging effect in H9c2 cells. Taken together, these results suggest that the antioxidant activity of the phenolic compounds ferulic acid, pterostilbene, and tyrosol does not appear to be directly involved in their cardioprotective effect against severe ER stress, suggesting the existence of alternative molecular mechanisms.

### 2.6. Ferulic Acid, Pterostilbene, and Tyrosol Reduce ER-Stress-Induced Activation of the PERK Pathway of the UPR

**Figure 7:**
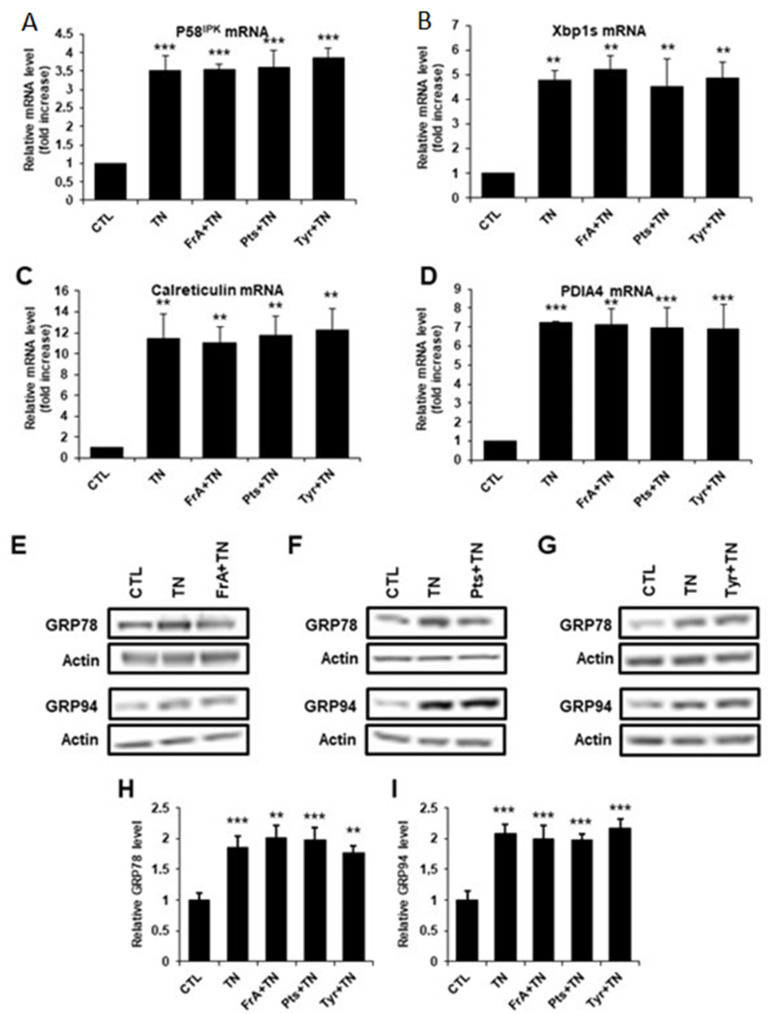
Ferulic acid, pterostilbene, and tyrosol do not modulate the IRE1 or the ATF6 pathway of the UPR in response to severe ER stress. H9c2 cells were pre-treated for 1 h with ferulic acid (FrA, 5 µM), pterostilbene (Pts, 0.5 µM), or tyrosol (Tyr, 1 µM), and then wer3e treated for 6 h with tunicamycin (TN, 10 µg/mL), and the relative mRNA levels of the IRE1 pathway target genes P58^IPK^ (**A**, *n* = 4) and Xbp1s (**B**, *n* = 4), and the ATF6 pathway target genes Calreticulin (**C**, *n* = 4) and PDIA4 (**D**, *n* = 3), were quantified by qPCR. The protein levels of the GRP78 and GRP94 chaperones were analyzed by western blot after 36 h. Representative western blots under ferulic acid (**E**), pterostilbene (**F**), or tyrosol (**G**) treatment are presented. Bar graphs show the quantitative western blot analysis for the GRP78 (**H**, *n* = 5) and GRP94 (**I**, *n* = 5) proteins. Actin was used as a loading control. Values represent mean ± S.E.M. ** *p* < 0.01, *** *p* < 0.001 vs. control.

**Figure 8:**
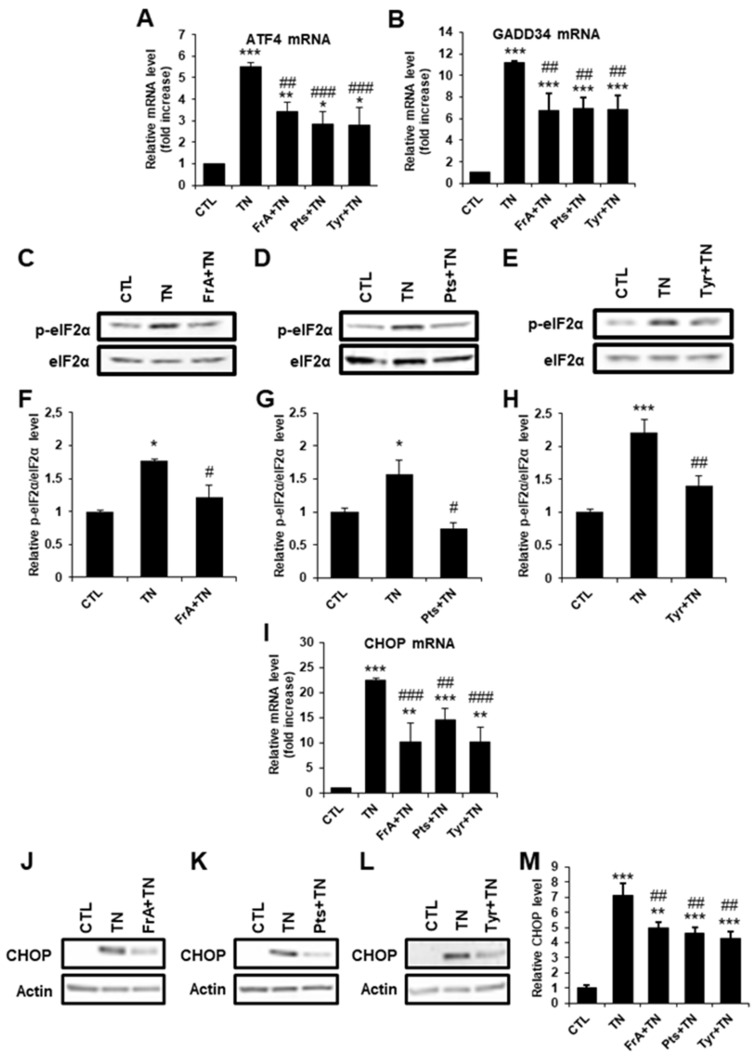
Ferulic acid, pterostilbene, and tyrosol modulate PERK pathway activation in response to severe ER stress. H9c2 cells were pre-treated for 1 h with ferulic acid (FrA, 5 µM), pterostilbene (Pts, 0.5 µM), or tyrosol (Tyr, 1 µM) and then were treated for 6 h with tunicamycin (TN, 10 µg/mL), and the relative mRNA levels of PERK pathway genes ATF4 (**A**, *n* = 4) and GADD34 (**B**, *n* = 5) were quantified by qPCR. The phosphorylation of the translation initiation factor eIF2α was analyzed by western blot after 1h30 treatment with TN ± phenolic compounds. Representative western blots under ferulic acid (**C**), pterostilbene (**D**), or tyrosol (**E**) treatment are presented. Bar graphs show the ratios of phosphorylated vs. total eIF2α (**F**–**H**, *n* = 4). The mRNA and protein levels of the pro-apoptotic factor CHOP were analyzed in response to TN with or without phenolic compounds (**I**–**M**, *n* = 5). Actin was used as a loading control. Values in the bar graphs represent mean ± S.E.M. * *p* < 0.05, ** *p* < 0.01 *** *p* < 0.001 vs. control; # *p* < 0.05, ## *p* < 0.01, ### *p* < 0.001 vs. TN.

To better understand the mechanisms of cardioprotection of ferulic acid, pterostilbene, and tyrosol against severe ER stress, we investigated whether these phenolic compounds could modulate the UPR response by measuring the activation of the three main pathways of the UPR, namely the IRE1, ATF6, and PERK pathways. TN increased the level of all the markers of the UPR tested both at the mRNA and protein levels (Figure 7 and Figure 8), indicating that the three pathways of the UPR were activated in our model. Following TN treatment, ferulic acid, pterostilbene, and tyrosol failed to modulate P58^IPK^ and Xbp1s mRNA levels (Figure 7A,B) as well as Calreticulin and PDIA4 mRNA levels (Figure 7C,D), which are markers of the IRE1 and ATF6 pathways, respectively. At the protein level, the ER chaperones GRP78 and GRP94, mostly regulated by the ATF6 and IRE1 pathways, were upregulated in response to TN, but ferulic acid, pterostilbene, and tyrosol did not modify their expression (Figure 7E–I). Thus, TN-mediated activation of the ATF6 and IRE1 pathways is not directly modulated by these molecules. By contrast, in response to TN, ferulic acid, pterostilbene, and tyrosol decreased the mRNA levels of ATF4 and GADD34 (Figure 8A,B), two markers of the PERK pathway. We also analyzed the phosphorylation level of the translation initiation factor eIF2α, which, when phosphorylated by the PERK kinase during ER stress, induces ATF4 and GADD34 expression. As revealed by western blot analysis, the TN-induced increase in eIF2α phosphorylation was significantly reduced by ferulic acid, pterostilbene, or tyrosol treatment (Figure 8C–H). Since the PERK/eIF2α/ATF4/CHOP signaling pathway is considered to play a key role in inducing cell apoptosis, the mRNA and protein levels of the pro-apoptotic transcription factor CHOP were analyzed. In response to TN alone, a strong increase in CHOP expression was observed (Figure 8I–M). When ferulic acid, pterostilbene, or tyrosol were added, both the mRNA and protein levels of CHOP were significantly lowered (Figure 8I–M), indicating that these phytochemicals decrease the activation of the pro-apoptotic arm of the UPR. Taken together, these results suggest that ferulic acid, pterostilbene, and tyrosol protect cardiomyocytes from apoptosis induced by severe ER stress by selectively reducing the activation of the PERK pathway.

### 2.7. Ferulic Acid, Pterostilbene, and Tyrosol Protect Cardiomyocytes from ER-Stress-Induced Apoptosis by Regulating the PERK Pathway through SIRT1-Mediated Deacetylation of eIF2α

**Figure 9:**
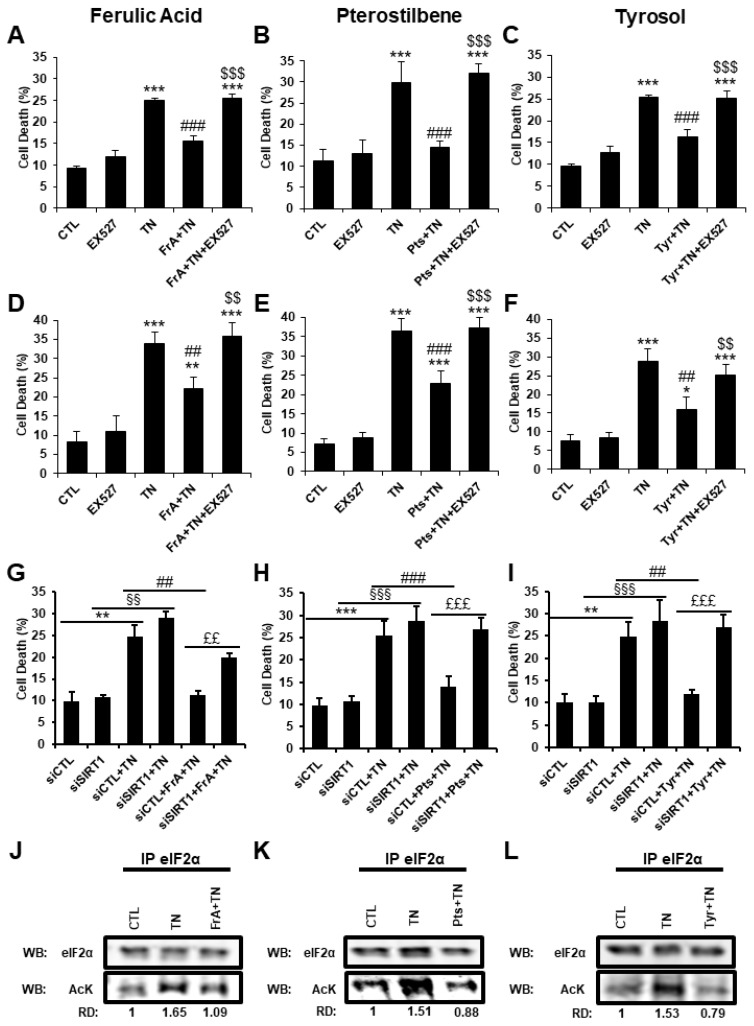
Ferulic acid, pterostilbene, and tyrosol protect cardiomyocytes from ER-stress-induced cell death through SIRT1-mediated deacetylation of eIF2α. ARVM and H9c2 cells were pre-treated for 1 h with ferulic acid (FrA, 5 µM), pterostilbene (Pts, 0.5 µM), or tyrosol (Tyr, 1 µM) with or without SIRT1 inhibitor EX-527 (5 µM), and were treated with tunicamycin (TN, 10 µg/mL). Cell viability was analyzed using FDA assay after 24 h for ARVM (**A**–**C**, *n* = 5) or 48 h for H9c2 cells (**D**–**F**, *n* = 4). FDA is cleaved into green fluorescein by intracellular esterases present in living cells. All non-FDA-fluorescing cells are considered dead. The percentages of dead cells are presented (Cell death (%)). H9c2 cells were transfected for 24 h with control or SIRT1 siRNAs, pre-treated for 1 h with ferulic acid (**G**, *n* = 4), pterostilbene (**H**, *n* = 6), and tyrosol (**I**, *n* = 6), and treated for 48 h with TN before analysis of cell viability by flow cytometry. Acetylation level of eIF2α on lysine residues was determined from anti-acetyl-lysine immunoprecipitates of H9c2 cells treated for 1h30 with TN with or without phenolic compounds. The ratio of acetylated vs. total eIF2α is presented relative to CTL. RD: relative density (**J**–**L**). Values in the bar graphs represent mean ± S.E.M. * *p* < 0.05, ** *p* < 0.01, *** *p* < 0.001 vs. control or siCTL; ## *p* < 0.01, ### *p* < 0.001 vs. TN or siCTL + TN; $$ *p* < 0.01, $$$ *p* < 0.001 vs. phenolic compounds + TN; §§ *p* < 0.01, §§§ *p* < 0.001 vs. siSIRT1; ££ *p* < 0.01, £££ *p* < 0.001 vs. siCTL + phenolic compounds + TN.

Ferulic acid, pterostilbene, and tyrosol were all described as potential SIRT1 activators in the literature [17,28]. In addition, we have previously demonstrated that SIRT1 protects the heart from severe ER stress by regulating the PERK pathway of the UPR through deacetylation of eIF2α on lysine K141/K143 [10]. We therefore investigated whether the cardioprotection afforded by ferulic acid, pterostilbene, or tyrosol could involve the modulation of the PERK/eIF2α pathway through SIRT1 activation. First, to decipher whether SIRT1 is involved in the cardioprotective effects of these phytochemicals against severe ER stress, cell death was measured in adult cardiomyocytes (ARVM) and H9c2 cells in response to TN ± phenolic compounds in the absence or the presence of EX-527, a selective pharmacological inhibitor of SIRT1. As shown in Figure 9, the inhibition of SIRT1 by EX-527 totally abrogated the protection afforded by ferulic acid, pterostilbene, and tyrosol against ER-stress-induced cell death both in ARVM (Figure 9A–C) and H9c2 cell (Figure 9D–F). Similar experiments were conducted in H9c2 cells, replacing EX-527 by siRNA targeting SIRT1 to avoid possible off-target effects of this pharmacological inhibitor. SIRT1 expression was knocked down by approximately 65% by SIRT1 siRNA in comparison to the negative control siRNA (Supplemental Figure S2). As revealed by flow cytometry experiments, ferulic acid, pterostilbene, and tyrosol decreased TN-induced cell death in H9c2 cells transfected with the control siRNA, but not in cells transfected with SIRT1 siRNA (Figure 9G–I), corroborating the results obtained with EX-527. These results indicate that SIRT1 plays a key role in the cardioprotection provided by ferulic acid, pterostilbene, or tyrosol in response to severe ER stress. Next, we tested whether eIF2α deacetylation was involved in the SIRT1-mediated cardioprotection conferred by the phytochemicals. Since antibodies directed against the acetylated form of eIF2α are not currently available, eIF2α was immunoprecipitated, and western blot were realized with an antibody which recognizes proteins acetylated on lysine residues. This approach has allowed us to successfully discover that eIF2α is acetylated on lysine K141 and K143 in cardiac cells in response to ER stress [10]. Figure 9J–L show that TN-induced ER stress increased the acetylation level of eIF2α. When ferulic acid, pterostilbene, or tyrosol were added, the level of acetylation of eIF2α was reduced in response to TN. Taken together, these results suggest that, in response to severe ER stress, the phenolic compounds tested herein promote SIRT1-mediated deacetylation of eIF2α to regulate the activation of the PERK pathway of the UPR.

## 3. Discussion

**Figure 10:**
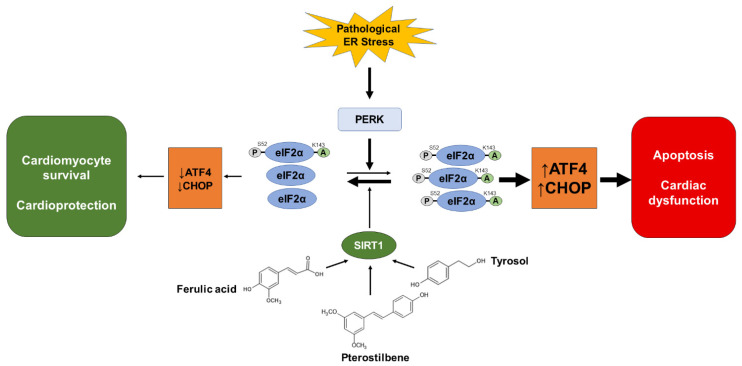
Simplified scheme illustrating the mechanism by which ferulic acid, pterostilbene, and tyrosol protect cardiac cells from adverse effects of ER stress. These phenolic compounds protect cardiac cells from ER stress-induced apoptosis by regulating the PERK pathway of the UPR through SIRT1-mediated deacetylation of the translation initiation factor eIF2α.

Over the past 25 years, ER stress has emerged as an important mechanism involved in the development and progression of multiple human diseases, specifically cardiac diseases [3]. In the field of cardiovascular research, it is recognized that a mild-to-moderate ER stress is beneficial for heart function as it promotes adaptative and compensatory mechanisms through the UPR, whereas a severe ER stress, associated with a hyperactivation of the UPR, is detrimental and promotes cardiomyocyte apoptosis, contributing to the development of cardiac dysfunctions [3,29,30]. Thus, cardiac therapy based on ER stress modulation is emerging as a promising new approach to promote beneficial adaptations and avoid apoptosis. In a previous article, we have shown that the deacetylase SIRT1 protects the heart from severe ER stress by specifically regulating the PERK pathway of the UPR through eIF2α deacetylation [10], suggesting that activation of SIRT1 could be an attractive strategy to modulate the UPR in order to avoid the detrimental effects of ER stress. The main focus of this study was to investigate the ability of ten phenolic compounds described in the literature as potential SIRT1 activators to protect the heart from severe ER stress. We found that ferulic acid, pterostilbene, and tyrosol were able to protect cardiomyocytes from ER-stress-induced apoptosis and to inhibit ER stress-induced cardiac dysfunction in vivo. Additionally, we demonstrated that these phenolic compounds are cardioprotective in response to severe ER stress by selectively regulating the PERK pathway of the UPR through activation of SIRT1-mediated deacetylation of the translation initiation factor eIF2α (Figure 10).

The beneficial effects of dietary phenolic compounds in preventing the development of cardiovascular diseases have been extensively studied in recent years. Their biological effects were commonly attributed to their antioxidant activity, which include direct reactive oxygen species’ scavenging activity. However, more recent analyses revealed that the cardioprotective effects of phenolic compounds could not be directly linked to their antioxidant activity in humans, and that other effects may be responsible for their positive cardiovascular influence [31,32]. For example, the anti-inflammatory, antithrombotic, and vasodilatory activities of phytochemicals and their ability to activate various cellular targets were proposed to be part of their beneficial effects on the cardiovascular system [33,34]. Our results suggest that the cardioprotective effect of ferulic acid, pterostilbene, and tyrosol against severe ER stress is not related to their antioxidant activity but rather to their ability to activate SIRT1.

Among the complex phenolic compounds’ superfamily, resveratrol is the most studied phytonutrient and the first molecule described as a natural SIRT1 activator [14]. Many studies showing the beneficial effects of this molecule in response to cardiac stresses [35] or ER stress [36] were carried out over the past 20 years. Aligning with reports linking the cardioprotective effect of resveratrol to the alleviation of ER stress [37,38,39], we show that resveratrol protects cardiomyocytes by decreasing ER-stress-induced apoptosis. Owing to the large number of studies showing the benefits of this stilbene in various contexts and diseases, different clinical trials have been conducted to assess the effectiveness of resveratrol for the treatment of human diseases, including ischemic and non-ischemic heart failure. Even if the results of the two randomized, double blinded, placebo-controlled studies REV-HF (Evaluating the Clinical Efficacy of REsVeratrol in Improving Metabolic and Skeletal Muscle Function in Patients with Heart Failure, clinicaltrials.gov NCT03525379) and RES-HF (RESveratrol: a Potential Anti-remodelling Agent in Heart Failure, clinicaltrials.gov NCT01914081) are still unknown, most clinical studies performed to date have shown insufficient results [40]. This is likely due to the poor bioavailability of resveratrol [41,42], its rapid elimination in urine [43], and its metabolization by the intestine and liver [44,45]. Therefore, resveratrol, the first-in-class among clinically tested polyphenols, does not appear to be the best candidate for the treatment of cardiac pathologies associated with ER stress due to unfavorable pharmacokinetics parameters. Here, we compared the cardioprotective effects of ferulic acid, pterostilbene, and tyrosol with those of resveratrol against severe ER stress, as they were also shown to activate SIRT1 in different cell types [15,17,28,46]. In adult rat cardiomyocytes (ARVM), the maximum protection afforded by each of these molecules against ER-stress-induced cell death was comparable or superior (tyrosol) to that offered by resveratrol (Figure 3). However, the concentrations of pterostilbene and tyrosol required to obtain such protection were respectively 5- and 2.5-fold lower than the required concentration of resveratrol, suggesting that pterostilbene and tyrosol are more potent phytonutrients to protect cardiac cells from severe ER stress. Pterostilbene is a natural analogue of resveratrol with two more *methoxy* groups on the A-benzene ring, which renders it more lipophilic, leading to better cellular uptake and oral absorption, which could account for its higher cardioprotective efficiency against severe ER stress [47]. In addition, in vivo studies have shown that, compared to resveratrol, pterostilbene exhibits a 7.5-times-higher half-life, a 4-times-higher oral bioavailability, a lower body clearance, and a better distribution to tissues, making this phytonutrient more suitable for use in clinical trials [48]. Despite its weak antioxidant activity [49], tyrosol is a more stable compound, and its significant intracellular accumulation might explain its more potent protective effects [50]. Finally, ferulic acid has been shown to be easily absorbed and to stay longer in the blood than the other phenolic compounds, properties that might provide therapeutic benefits for the treatment of cardiac diseases associated with ER stress [51].

In this study, we also investigated the mechanisms underlying cardioprotection by phytochemicals treatment. We provide evidence that in response to ER stress, ferulic acid, pterostilbene, and tyrosol protect cardiomyocytes from severe ER stress by selectively downregulating the PERK pathway of the UPR. In response to ER stress, the PERK-mediated phosphorylation of the translation initiation factor eIF2α on serine 51/52 inhibits general protein translation initiation and allows for the selective translation of specific mRNAs, including that which encode the transcription factor ATF4, which increases the transcription of GADD34 and the pro-apoptotic transcription factor CHOP [52]. Our results demonstrate that ferulic acid, pterostilbene, and tyrosol decrease eIF2α phosphorylation, as well as the transcription of ATF4 and GADD34. Additionally, these phenolic compounds also decrease the expression of CHOP both at the mRNA and protein levels. CHOP is the main executioner of the pro-apoptotic arm of the UPR, which triggers the mitochondrial pathway of apoptosis through regulation of the expression of the members of the Bcl-2 family [53]. Therefore, our data suggest that ferulic acid, pterostilbene, and tyrosol, by downregulating the PERK/eIF2α/ATF4 pathway, reduce the level of CHOP expression and consequently reduce the level of ER-stress-induced apoptosis in cardiac cells. It should be noted that the PERK pathway is not completely inhibited by these molecules since neither the downregulation of ATF4, GADD34, and CHOP, nor the phosphorylation of eIF2α, were complete, suggesting that the beneficial responses mediated by a moderate activation of the PERK pathway may still occur (Figure 10).

Since ferulic acid, pterostilbene, and tyrosol were suggested to activate SIRT1 [15,17,28,46], we examined whether SIRT1 was involved in the cardioprotective effects of these phenolic compounds against ER-stress-induced cell death. When SIRT1 was inhibited by the specific compound EX-527 or knocked down by siRNA, the protective effects of ferulic acid, pterostilbene, and tyrosol were lost. These results indicate that SIRT1 plays an important role in the protection afforded by these phytochemicals in response to severe ER stress in cardiac cells. These findings echo our previous studies showing that SIRT1 protects the heart from severe ER stress by regulating the PERK pathway of the UPR through deacetylation of eIF2α on lysines K141/K143 [10]. Of note, our previous results also indicated that in parallel to its acetylation, the level of eIF2α phosphorylation was increased upon ER stress when SIRT1 was inhibited or deleted, suggesting a dynamic interplay between these two post-translational modifications. Here, we show that the activation of SIRT1 by ferulic acid, pterostilbene, or tyrosol decreases the acetylation of eIF2α as well as its phosphorylation. Although further studies are needed to better understand the interconnection between these two post-translational modifications, we propose that ferulic acid, pterostilbene, and tyrosol, through the SIRT1-mediated deacetylation of eIF2α, decrease its level of phosphorylation, resulting in a reduced expression of ATF4 and of the pro-apoptotic factor CHOP, leading to less apoptosis. Therefore, the ferulic-acid-, pterostilbene-, or tyrosol-induced activation of SIRT1, by decreasing the level of activation of the PERK pathway, allow for a better survival of cardiac cells in response to ER stress. Different studies have shown a significant protection conferred by these natural molecules in other contexts of cardiac stresses that was attributed to their antioxidant, anti-inflammatory, antithrombotic, and vasodilatory activities [54]. Thus, it would be interesting to investigate whether SIRT1-mediated regulation of the PERK pathway is involved in the cardioprotection conferred by ferulic acid, pterostilbene, and tyrosol in other pathophysiological models of cardiovascular pathologies.

## 4. Materials and Methods

### 4.1. Animals and Treatments

Based on the literature, adult male C57BL/6J mice were given i.p. injection of ferulic acid, pterostilbene or tyrosol at 25 mg/kg body weight or the equivalent volume of vehicle (NaCl 0.9%) every day during 5 days. At day 3, mice were given i.p. injection of tunicamycin (TN) 2 mg/kg body weight or the equivalent volume of vehicle (Dextrose 150 mM) to induce severe ER stress as previously reported [6,10]. Echocardiographic parameters were assessed at day 5, 2 h after the last phytochemical injection. All experiments were performed in conformity with the European Community guiding principles on the care and use of animals (EU directive 2010/63/EU for animal experiments). Authorizations to conduct animal experiments were obtained from the French Ministère de l’Enseignement Supérieur, de la Recherche et de l’Innovation (n°B9201901, 3 November 2015).

### 4.2. Echocardiography

Transthoracic echocardiography was performed using a 15 MHz transducer under 2% isoflurane gas anesthesia. Two-dimensional-guided (2D) M-mode echocardiography was used to determine the left ventricular chamber volume at systole and diastole and contractile parameters such as ejection fraction (EF).

### 4.3. Cell Culture and Reagents

The H9c2 rat cardiomyoblast cell line was purchased from ATCC (n° CRL-1446, Manassas, VA, USA). Cells were cultured in DMEM medium with 1 mM pyruvate and 4.5 g/L glucose (Fisher Scientific, Illkirch, France) and supplemented with 100 U/mL penicillin, 100 mg/mL streptomycin (Fisher Scientific, Illkirch, France), and 10% FBS (Corning, Glendale, AZ, USA) at 37 °C under 5% CO_2_/95% air. Severe ER stress was induced by TN treatment at 10 µg/mL (Tocris, Bristol, UK). EX-527 Sirtuin-1 inhibitor was used at 5 µM (Tocris, Bristol, UK). Its IC50 value was determined using the in vitro Fluor de Lys deacetylation assay and a purified human SIRT1 range from 38 to 150 nM. Berberine, Catechin, Ferulic Acid, Malvidin, Tyrosol, and Resveratrol were purchased from Merck-Sigma (Saint-Quentin-Fallavier, France). Butein, Isoliquiritigenin, Piceatannol, and Pterostilbene were purchased from Cayman (Ann Arbor, MI, USA). All phenolic phytochemicals were diluted in DMSO.

### 4.4. Flow Cytometry

Cell viability was assessed by using a fluorescein diacetate (FDA; Merck-Sigma, Saint-Quentin-Fallavier, France) fluorescent probe. After 48 h of treatment, cells were trypsinized and incubated for 10 min at 37 °C with 0.2 µg/mL FDA and analyzed by flow cytometry. Cell size was assessed based on FSC and SSC patterns. Plasma membrane integrity was estimated by adding 10 µg/mL of propidium iodide (PI) (Merck-Sigma, Saint-Quentin-Fallavier, France) just before flow cytometric analysis. DNA fragmentation (Sub G1 population) was analyzed by measuring DNA content. Briefly, after 48 h of treatment, cells were trypsinized and fixed in 70% ethanol during 24 h at −20 °C. Cells were then incubated overnight at 4 °C in dark with 50 µg/mL PI and 250 µg/mL RNAse A (Merck-Sigma, Saint-Quentin-Fallavier, France) and analyzed by flow cytometry. MitoSOX™ Red was used to measure the relative levels of the mitochondrial superoxide anion O_2_^−^. Once in the mitochondria, MitoSOX™ Red reagent is oxidized by superoxide anions and exhibits red fluorescence detected by flow cytometry. CM-H2DCFHDA was used to measure cellular H_2_O_2_ levels. After 24 h of treatment, cells were centrifuged, washed with PBS, and incubated with 2 μM of MitoSOX™ Red for 10 min or with 1 µM CM-H2DCFHDA for 15 min at 37 °C and then analyzed by flow cytometry. A FC500 flow cytometer (Beckman Coulter, Roissy, France) was used for the analyses.

### 4.5. Isolation of Adult Rat Ventricular Myocytes (ARVM)

Adult Wistar male rats (250–300 g) were anesthetized by intraperitoneal injection of Dolethal (150 mg/kg), and the beating hearts were rapidly collected after dissection and put in a cold Ca^2+^-free Ringer solution. All animal experimental procedures were approved by the animal ethics committee of Paris-Saclay University, authorized by the French government (Authorization Number: B9201901), and complied with directive 2010/63/EU of the European Parliament on the protection of animals used for scientific purposes. Hearts were then perfused retrogradely through the ascending aorta according to the Langendorff method and washed for 5 min at a constant flow of 6 mL/min with Ca^2+^-free Ringer solution at 37 °C with oxygen. Enzymatic dissociation was performed by perfusing during 45 min at a constant flow of 4 mL/min with Ringer solution with 1 mg/mL collagenase A (Merck-Sigma, Saint-Quentin-Fallavier, France) and 20 µM of free Ca^2+^ adjusted following 300 µM EGTA at 37 °C with oxygen. Ventricles were then separated from atria, finely chopped, and gently agitated to dissociate individual cells. Cell suspension was then filtered and the cells were allowed to settle down. The supernatant was discarded to eliminate dead cells and debris and resuspended during 5 min in Ca^2+^-free Ringer solution containing a progressively increasing calcium concentration. Cells were plated into dishes coated with laminin on DMEM medium with 4.5 g/L glucose and 1 mM pyruvate supplemented with 100 U/mL penicillin, 100 mg/mL streptomycin. After 2 h of plating, the medium was changed and cardiomyocytes were treated for 24 h.

### 4.6. Evaluation of Adult Cardiomyocyte Death by Fluorescence Microscopy

To analyze cell death, adult rat ventricular myocytes (ARVM) were stained with 0.2 µg/mL of FDA (Merck-Sigma, Saint-Quentin-Fallavier, France) for 10 min. FDA-negative cardiomyocytes were considered as dead cells. More than 1000 cells were counted for each condition on a Leica fluorescence microscope (Leica Microsystems, Nanterre, France), and results were expressed as a percentage of FDA-negative cells.

### 4.7. RNA Isolation and Quantitative RT-PCR

RNA was isolated from cultured cells treated during 6 h with or without TN and phytochemicals, using TRI Reagent^®^ (MRCgene, Cincinnati, OH, USA) according to the manufacturer’s instructions. Total RNA (1 µg) was reverse transcribed using a BioRad iScript reverse transcription kit. Real-time PCR was performed using the SYBR^®^ green method on the CFX96 Touch^TM^ Real-Time PCR Detection system (Bio-Rad, Marnes-la-Coquette, France) from 2.5 ng cDNA. mRNA levels for all target genes were normalized to Ywhaz and RPL32. The PCR primers were obtained from Eurofins and are listed in supplemental Table S1. The results were quantified according to the Cq value method, where Cq is defined as the quantification cycle of PCR at which the amplified product is detected. The ratio (1+Etargetgene)ˆ-(Cqsample-Cqcontrol)targetgene/(1+Ereferencegene)ˆ-(Cqsample-Cqcontrol) gene was calculated, where E represented the efficiency of the quantitative PCR reaction. With this calculation, the expression of the control was equivalent to one.

### 4.8. Western Blot Analysis

Cells were lysed in a RIPA lysis buffer (50 mM Tris-base pH 8, 150 mM NaCl, 1% Triton, 1 mM EDTA, 0.1% SDS, 0.5% deoxycholic acid) supplemented with cocktails of proteases (N°539134, Merck-Sigma, Saint-Quentin-Fallavier, France), phosphatases (N°524629, Merck-Sigma, Saint-Quentin-Fallavier, France), and deacetylase inhibitors (sc362323, Santa-Cruz Biotechnology, Dallas, TX, USA) for 30 min at 4 °C. Proteins (25 µg) were separated by 4–20% Tris-Glycine gel electrophoresis and transferred to PVDF membranes (Fisher scientific). Membranes were incubated overnight at 4 °C with the following antibodies: anti-GRP78, anti-GRP94, and anti-eIF2α from Cell Signaling Technology (#3183, #2104 and #2103), anti-CHOP from Genetex (GTX11419), anti-Phospho-eIF2α from Invitrogen (44728G), and anti-Actin from Santa Cruz (sc-47778). Proteins were detected on an iBright FL1000 Imager (ThermoFischer Scientific, Illkirch, France) by using the ECL method according to the manufacturer’s instructions (Fisher Scientific, Illkirch, France). To determine the working range of each antibody, different protein quantities, antibody concentrations, and times of exposure were tested. To calculate the relative density (RD), ImageJ software was used, and the intensity of each protein was normalized to actin. The data obtained were then expressed as the ratio of the intensity of the protein in treated cells to that of the corresponding protein in untreated cells.

### 4.9. siRNA Transfection

SIRT1 siRNA (sc-108043) and non-targeting control siRNA were purchased from Santa Cruz (sc-37007). H9c2 cells were plated in 24-well plates overnight at 35 × 10^4^ cells per well, and were transfected with 8 pmol of either non-targeting control siRNA or SIRT1 siRNA according to the manufacturer’s instructions. 24 h after transfection, the medium was removed, and cells were pre-treated or not for 1 h with ferulic acid, pterostilbene, or tyrosol and then incubated for 48 h with TN before analysis.

### 4.10. Protein Immunoprecipitation

To analyze the acetylation level of eIF2α, H9c2 cells were lysed in a denaturing IP buffer (1% SDS, 50 mM Tris-HCl, 5 mM EDTA, 10 mM DTT, 15 U/mL DNase, pH 7.4) supplemented with a cocktail of inhibitors of proteases, phosphatases, and deacetylases. Lysates were heated at 90 °C for 5 min and then diluted in a non-denaturing buffer to trap SDS (1% triton, 50 mM Tris HCl, 5 mM EDTA, 300 mM NaCl, pH 7.4), supplemented with protease, phosphatase, and deacetylase inhibitor cocktails. eIF2α was immunoprecipitated using an anti-eIF2α antibody coated on G magnetic beads (Dynabeads™, Fisher Scientific, Illkirch, France, 10007D). Immunoprecipitated proteins were run on an SDS-PAGE gel and were revealed with anti-eIF2α or anti-acetylated lysine antibodies (Cell Signaling Technology, Danvers, MA, USA, #2103 and #9681).

### 4.11. Statistical Analysis

All data were presented as means ± S.E.M. Data were analyzed using Sigma Stat (Sigma Stat, version 3.0, Systat Software, San Jose, CA, USA). One-way ANOVA was used to assess differences among groups, followed by Newman–Keuls post hoc tests. Differences between groups were considered significant if the *p*-value was * *p* < 0.05, ** *p* < 0.01, *** *p* < 0.001 versus the control or siCTL; # *p* < 0.05, ## *p* < 0.01, ### *p* < 0.001 versus TN or siCTL + TN; $ *p* < 0.05, $$ *p* < 0.01, $$$ *p* < 0.001 versus phytochemical + TN; § *p* < 0.05, §§ *p* < 0.01, §§§ *p* < 0.001 versus siSIRT1; £ *p* < 0.05, ££ *p* < 0.01, £££ *p* < 0.001 versus siCTL + phytochemical + TN; ¤ *p* < 0.05, ¤¤ *p* < 0.01, ¤¤¤ *p* < 0.001 versus H_2_O_2_.

## 5. Conclusions

Interestingly, the three phenolic compounds identified in our study as inhibitors of ER-stress-induced cardiac cell death are widely available in our diet. Indeed, ferulic acid is the most abundant polyphenol found in cereals and some fruits [55], pterostilbene is primarily found in grapes and blueberries [47], and tyrosol is abundantly found in olives and derivatives [56]. Our results demonstrated that these natural phenolic compounds protect the heart from adverse effects of ER stress by regulating the PERK pathway of the UPR through SIRT1-mediated deacetylation of the translation initiation factor eIF2α. Therefore, increasing SIRT1 deacetylase activity by these molecules or more potent/stable chemically modified derivatives of these phytonutrients might be a promising strategy for the treatment of cardiomyopathies associated with ER stress.

## Supplementary Materials

The following supporting information can be downloaded at: https://www.mdpi.com/article/10.3390/ijms23126628/s1.
