# Supplementary material for: Ferulic Acid, Pterostilbene, and Tyrosol Protect the Heart from ER-Stress-Induced Injury by Activating SIRT1-Dependent Deacetylation of eIF2α
Source: Int J Mol Sci. 2022 Jun 14;23(12):6628. doi: 10.3390/ijms23126628 (PMC9224298; doi:10.3390/ijms23126628)

Table S1. Sequences of qPCR primers used in this study.

Gene	Species	Forward primer	Reverse primer
ATF4	mouse/rat	AAACCTCATGGGTTCTCCAG	TCTCCAACATCCAACTGTCC
Calreticulin	mouse/rat	CTGGGTCGAATCCAAACATAA	GCGTAAAATCGGGCATCTT
CHOP	mouse/rat	TATCTCATCCCCAGGAAACG	CAGGGTCAAGAGTAGTGAAGGTTT
GADD34	mouse/rat	GGACCCTGAGATTCCTCTGA	GCCCAGACAGCAAGGAAAT
$P58^{IPK}$	mouse/rat	CAGTTTCATGCTGCCGTAGA	GCTTTTGATTTGCCCATAGC
PDIA4	mouse/rat	CTGATTGGACACCTCCACCT	AGGGGCAAGTTTCTTGCAG
Xbp1s	mouse/rat	TGCTGAGTCCGCAGCAGGTG	ACAGGGTCCAACTTGTCCAG

Table S2. Echocardiographic parameters of mice treated with ferulic acid, pterostilbene or tyrosol in response to ER stress.

	CTL	TN	FrA	FrA+TN	Pts	Pts+TN	Tyr	Tyr+TN
EF (%)	75.2 ± 1.6	57.8 ± 2.81***	75.69 ± 1.70	73.19 ± 2.03###	72.00 ± 1.31	76.69 ± 0.88###	73.01 ± 1.79	76.56 ± 2.46###
FS (%)	38.1 ± 1.54	25.98 ± 1.62***	38.69 ± 1.54	36.64 ± 1.64###	35.63 ± 1.01	39.51 ± 0.80###	36.43 ± 1.44	39.56 ± 2.14###
HR (bpm)	492 ± 17	448 ± 32	491 ± 17	494 ± 25	566 ± 36	455 ± 8	498 ± 20	482 ± 22
LViDd (mm)	3.49 ± 0.16	3.38 ± 0.16	3.35 ± 0.15	3.34 ± 0.20	3.49 ± 0.27	2.82 ± 0.13	3.24 ± 0.17	2.88 ± 0.23
LViDs (mm)	2.16 ± 0.14	2.49 ± 0.11	2.05 ± 0.04	2.12 ± 0.17	2.25 ± 0.18	1.70 ± 0.09	2.06 ± 0.11	1.74 ± 0.15
LVAWd (mm)	0.90± 0.05	0.79 ± 0.05	0.84 ± 0.03	0.82 ± 0.06	1.00 ± 0.10	0.93 ± 0.08	0.94 ± 0.08	0.96 ± 0.09
LVAWs (mm)	1.42 ± 0.12	1.10 ± 0.08	1.43 ± 0.08	1.34 ± 0.08	1.43 ± 0.18	1.44 ± 0.10	1.57 ± 0.08	1.43 ± 0.14
LVPWd (mm)	1.33 ± 0.13	1.01 ± 0.08	1.02 ± 0.13	1.04 ± 0.07	0.89 ± 0.05	1.28 ± 0.05	0.91 ± 0.12	0.97 ± 0.12
LVPWs (mm)	1.72 ± 0.17	1.20 ± 0.12	1.43 ± 0.08	1.43 ± 0.09	1.44 ± 0.12	1.64 ± 0.07	1.10 ± 0.10	1.41 ± 0.13
TWTd (mm)	2.21 ± 0.12	1.775 ± 0.13	1.86 ± 0.10	1.85 ± 0.09	1.90 ± 0.13	2.22 ± 0.10	1.85 ± 0.12	1.93 ± 0.20
TWTs (mm)	3.14 ± 0.17	2.26 ± 0.13	2.64 ± 0.04	2.78 ± 0.08	2.87 ± 0.22	3.07 ± 0.05	2.68 ± 0.05	2.84 ± 0.25

Figure S1

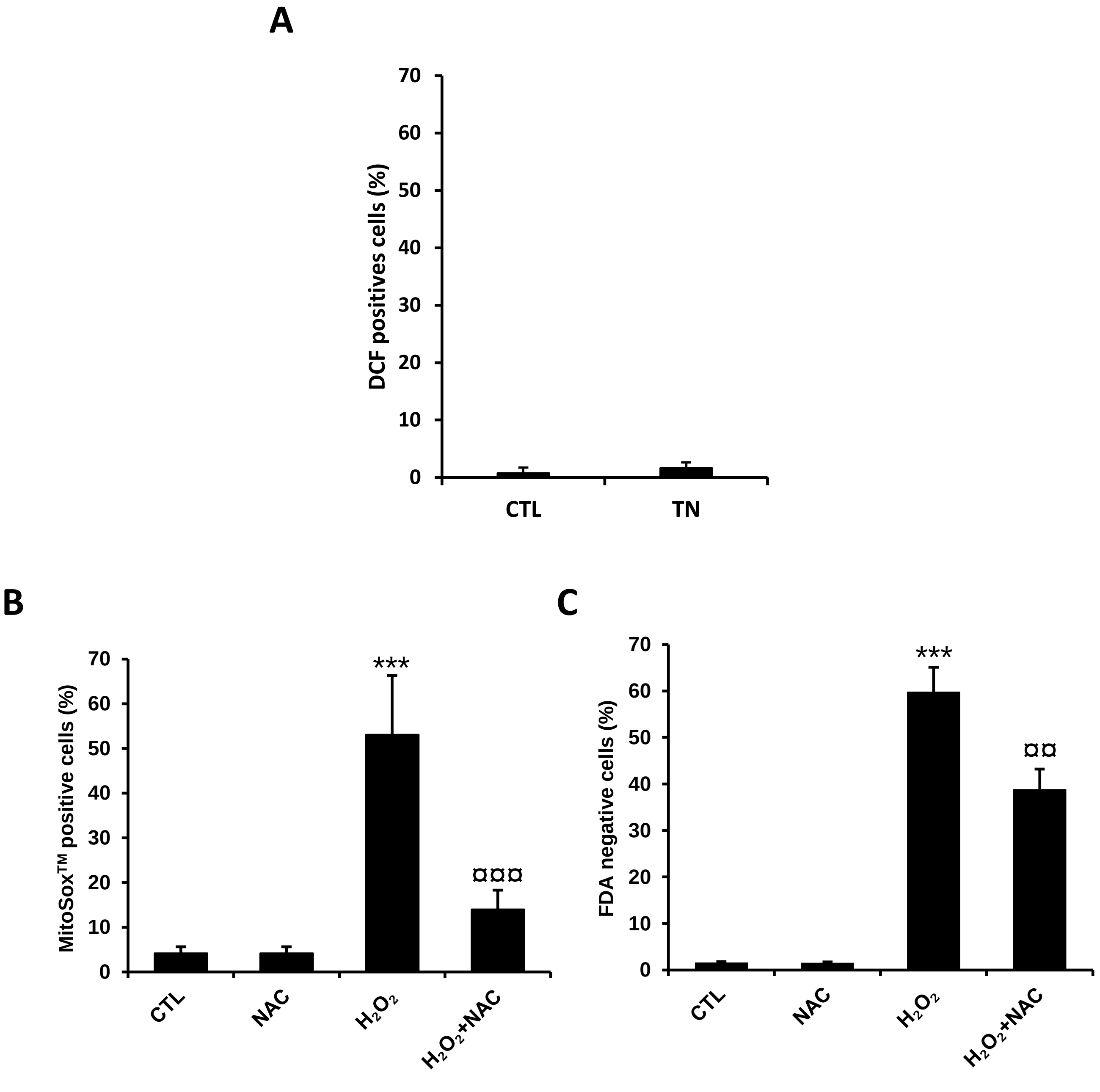
A
DCF positives cells (%)
70
60
50
40
30
20
10
0
CTL
TN
B
MitoSox™ positive cells (%)
70
60
50
40
30
20
10
0
***
¤¤¤
CTL
NAC
H2O2
H2O2+NAC
C
FDA negative cells (%)
70
60
50
40
30
20
10
0
***
¤¤
CTL
NAC
H2O2
H2O2+NAC

Figure S2

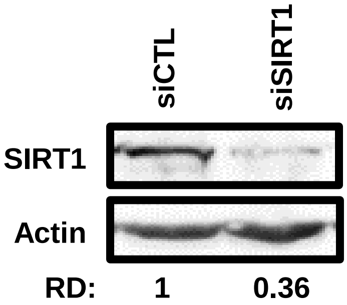

Supplement: Supplementary file 1 [file ijms-23-06628-s001.zip › ijms-1724124-supplementary.pdf]
